# Update on Myocarditis: From Etiology and Clinical Picture to Modern Diagnostics and Methods of Treatment

**DOI:** 10.3390/diagnostics13193073

**Published:** 2023-09-28

**Authors:** Ratko Lasica, Lazar Djukanovic, Lidija Savic, Gordana Krljanac, Marija Zdravkovic, Marko Ristic, Andjelka Lasica, Milika Asanin, Arsen Ristic

**Affiliations:** 1 Department of Cardiology, Emergency Center, University Clinical Center of Serbia, 11000 Belgrade, Serbia; lazardjukanovic08@gmail.com (L.D.); lidijasavic2007@gmail.com (L.S.); gkrljanac@yahoo.com (G.K.); masanin2013@gmail.com (M.A.); 2 Faculty of Medicine, University of Belgrade, 11000 Belgrade, Serbia; sekcija.kardioloska@gmail.com; 3 Department of Cardiology, University Medical Center Bezanijska Kosa, 11000 Belgrade, Serbia; 4 Department of Cardiology, University Clinical Center of Serbia, 11000 Belgrade, Serbia; marko.ris@live.com; 5 Health Center New Belgrade, 11000 Belgrade, Serbia; aleksandralasica763@gmail.com

**Keywords:** acute myocarditis, chronic inflammatory cardiomyopathy, heart failure, cardiac magnetic resonance, endomyocardial biopsy

## Abstract

Although the frequency of myocarditis in the general population is very difficult to accurately determine due to the large number of asymptomatic cases, the incidence of this disease is increasing significantly due to better defined criteria for diagnosis and the development of modern diagnostic methods. The multitude of different etiological factors, the diversity of the clinical picture, and the variability of the diagnostic findings make this disease often demanding both for the selection of the diagnostic modality and for the proper therapeutic approach. The previously known most common viral etiology of this disease is today overshadowed by new findings based on immune-mediated processes, associated with diseases that in their natural course can lead to myocardial involvement, as well as the iatrogenic cause of myocarditis, which is due to use of immune checkpoint inhibitors in the treatment of cancer patients. Suspecting that a patient with polymorphic and non-specific clinical signs and symptoms, such as changes in ECG and echocardiography readings, has myocarditis is the starting point in the diagnostic algorithm. Cardio magnetic resonance imaging is non-invasive and is the gold standard for diagnosis and clinical follow-up of these patients. Endomyocardial biopsy as an invasive method is the diagnostic choice in life-threatening cases with suspicion of fulminant myocarditis where the diagnosis has not yet established or there is no adequate response to the applied therapeutic regimen. The treatment of myocarditis is increasingly demanding and includes conservative methods of treating heart failure, immunomodulatory and immunospressive therapy, methods of mechanical circulatory support, and heart transplantation. The goal of developing new diagnostic and therapeutic methods is to reduce mortality from this complex disease, which is still high.

## 1. Introduction

Myocarditis is a disease of the heart muscle caused by inflammation, which is the most often the result of an infectious process, although it can also be caused by hypersensitivity to drugs, radiation, metabolic disorders, collagenoses, sarcoidosis, Kawasaki disease, and exposure to excessive heat or chemicals [1,2]. The prevalence of myocarditis in the general population is difficult to determine because a large number of patients remain undiagnosed, but it roughly ranges from 10.2 to 105.6 per 100,000 worldwide, and its annual occurrence is estimated at about 1.8 million cases [3]. A Swedish study by Fu M. and associates indicated an increase in the incidence of myocarditis from 6.3 to 8.6 per 100,000 from 2000 to 2014 [4]. This increase in incidence can be explained by better diagnosis of myocarditis and better defined criteria for diagnosing myocarditis. Autopsy studies have shown the presence of myocardial inflammation in 6–42% of autopsies after sudden cardiac death [5,6]. According to modern registries, myocarditis is a disease of the younger population, with the average age of patients being between 30 and 45, and is considered one of main causes of sudden cardiac death in young people. Myocardial disease is the cause of death in 25% of suddenly deceased patients under the age of 21, and in 20% of cases this disease is a precursor to dilated cardiomyopathy (DCM), which is the most common reason for heart transplantation today [7,8].

Special attention was paid to this disease by the global public during the outbreak of the SARS-CoV-2 virus pandemic, considering that as many as 28% of patients had myocardial damage accompanied by an increase in the value of cardio-specific troponin [9]. Data from a respective cohort study indicate that 54% of patients after confirmed COVID-19 infection had a cardiac magnetic resonance (CMR) finding that indicated myocarditis (myocardial edema or late gadolinium accumulation) [10]. There is evidence that the probability of occurrence of myocarditis is up to seven times higher in unvaccinated patients compared to patients who received the SARS-CoV-2 virus vaccine [11].

Men are two to four times more likely to suffer from myocarditis than women [12]. Affected men are more often younger than women, and the highest rate of occurrence of the disease in men is between 16 and 20 years of age, while women were the most affected at the ages of 56–60 [13,14,15].

Myocarditis is a very common disease in children, and due to the heterogeneity of the symptoms of the clinical picture, the diagnosis is made only in 17% of patients during the first examination [16]. Today, the incidence rate in children aged 0–15 years is estimated at 1.95/100,000 persons per year [17]. In the child population, myocarditis is most common in boys.

## 2. Materials and Methods

In the preparation of this paper, an electronic search was performed in reliable databases (PubMed, Web of Science, Scopus, Google Scholar) in order to identify all relevant reports on myocarditis. A comprehensive search strategy was developed by the authors based on the following keywords: acute myocarditis, chronic inflammatory cardiomyopathy, heart failure, cardiac magnetic resonance, endomyocardial biopsy.

## 3. Etiological Factors and Pathophysiological Mechanisms of Myocarditis

### 3.1. Infectious Causes of Myocarditis

Cardiotropic viruses are the most common causes of myocarditis, and lead to cardiomyocyte damage through direct cardiotoxic effects. These viruses primarily include enteroviruses (the Coxsackie A and Coxsackie B viruses, echoviruses) and adenoviruses [18]. Genetic predisposition can affect the increased susceptibility to infection caused by cardiotropic viruses and the development of the chronic course of the disease [19]. In addition to cardiotropic viruses, the causative agents of myocarditis are vasculotropic viruses (erythroviruses—parvovirus B19) and lymphotropic viruses (herpes viruses, Epstein Barr virus, Cytomegalovirus) [18]. Cytomegalovirus and Toxoplasma gondii are potential causes of myocarditis in heart transplant patients [20,21]. Viruses mostly act through the activation of the immune system and the appearance of a cytotoxic storm of immune mimicry [22]. Such viruses include human immunodeficiency virus, hepatitis C virus, and influenza A and B viruses, as well as SARS-CoV-1 and 2 viruses [18]. A special mechanism of action was discovered in the SARS-CoV-2 virus, which causes direct damage to cardiomyocytes by acting on Angiotensin II receptors [23,24]. Enteroviruses, which are one of the most common causes of viral myocarditis, bind to specific receptors on cardiomyocytes, multiply, and cell lysis follows. In about 50% of these patients, there is a spontaneous clinical recovery, while the persistence of the virus genome in the myocardium most often leads to left ventricular dysfunction and a worse clinical outcome [25]. Today, with the development of molecular technology, other viral agents of acute myocarditis (AM) such as PVB19 and HHV61 have emerged [26,27]. PVB19 is a virus that can cause both virus-mediated and virus-induced myocarditis. In children, PVB19 causes systemic infection associated with AM where PVB19 can be detected in both plasma and myocardium [28].

There are two mechanisms of myocardial damage during viral infection:

(1) Direct cytotoxic effect (Murine model: virus penetration into myocytes, their binding to a specific receptor, virus replication, and consequent necrosis of myocites), binding to intracellular agents (myosin), and activation of the immune system characterized by the invasion of natural killer cells and macrophages accompanied by T lymphocytes.
(2) Secondary phase (autoimmune reaction).

After the acute phase of viral-mediated myocarditis, there are three generally accepted clinical possibilities: (1) the virus clears without residual inflammation, resulting in complete healing; (2) viral infection persists with or without inflammation; or (3) viral infection results in autoimmune-mediated inflammation that persists despite viral clearance [29]. If the infectious agent is quickly eliminated and the inflammatory process ends, the disease will be cured with only minor changes in myocardium. If irreversible myocardial damage has developed, the clinical picture evolves into DCM [29].

Cytokine release syndrome (CRS) is defined as an inflammatory response that leads to the release of large amounts of cytokines in the body in response to infection or drug administration [30]. While mild CRS may be self-limited, presenting with flu-like symptoms and fever, severe CRS may be accompanied by hypoxia and left ventricular dysfunction. Hemodynamic instability associated with CRS can be accompanied by hypotension and rhythm disturbances [31]. The literature describes cases of myocarditis caused by cytokine storms, especially in patients with COVID-19 infection [32].

Infection with the human immunodeficiency virus (HIV) is a global problem. A large percentage of patients with HIV infection suffer from myocarditis, although the incidence of myocarditis associated with HIV infection has been declining in recent years [33]. In contrast, the incidence of heart failure (HF) is increasing, primarily due to HF with preserved ejection fractions [34,35]. Today, the estimated incidence of DCM associated with HIV disease is 1.6% [36]. It has been shown that there is no clear association between HIV infection and cardiomyocyte dysfunction. However, it has been shown that the presence of the virus can condition the appearance of an inflammatory response and the activation of cytokines and consequent damage to cardiomyocytes [37]. An additional important factor in the occurrence of myocarditis and DCM associated with HIV infection are opportunistic infections [38]. Of course, the toxic effects of ARV therapy cannot be ignored either [39]. According to autopsy data published before the ART era, myocarditis was diagnosed in 40–52% of patients who died of AIDS [40].

Although in patients with myocarditis the viral etiology of the disease is always thought of first, other infectious agents should also be considered. In patients with myocarditis in whom we have information about a tick bite in the epidemiological anamnesis, one must always think about the existence of Lyme disease and anaplasmosis. Lyme disease must always be considered in case of conduction disorders of unknown origin. The incidence of cardiac involvement in Lyme disease is between 0.3–10% [41]. In the pediatric population, that percentage is much higher and reaches up to 30% of cases [42]. Although much less often, cases of myocarditis associated with anaplasmosis have also been described [43,44]. A detailed travel history must be taken to assess the possible risk of schistosomiasis (primarily in Africa, but also in Japan, China, the Middle East, South America, the Caribbean), Chagas disease (North America, Europe, Japan, Australia), tuberculosis (Asia and Africa, Eastern Europe), or Hepatitis C (Japan). In endemic areas, the presence of dengue fever must also be considered [3,45].

### 3.2. Eosinophilic Myocarditis

Eosinophilic myocarditis (EM) is characterized by the presence of eosinophilic infiltration in myocardial tissue. EM may be accompanied by a hematologic disorder (hypereosinophilic syndrome (idiopathic form) or the myeloproliferative form of the disease) characterized by persistent eosinophilia (>1.5 × 10^9^/L for more than six months) and organ damage [46,47]. Approximately 20% of patients with this syndrome have myocardial involvement [48]. EM has been observed to be associated with hypersensitivity reactions to chemicals and some drugs (b-lactam antibiotics, clozapine, carbamazepine, minocycline, etc.) [49,50,51,52]. Sometimes parasitic infections and malignancies can be associated with EM [53,54]. EM can occur with some systemic diseases such as the association of eosinophilic granulomatosis with polyangiitis [55].

Among the causes of eosinophilic myocarditis, DRESS myocarditis is the most common and severe. Adverse drug reactions accompanied by eosinophilia and systemic symptoms (DRESS syndrome) represent an idiosyncratic and life-threatening drug reaction [50,56]. The most common drugs that can lead to this form of myocarditis are minocycline (19%) and allopurinol (12%) [51,56,57]. Usually, symptoms appear a few weeks to a few months after oral administration of the drug [Japanese]. This syndrome is characterized by fever, lymphadenopathy, liver dysfunction, and systemic skin rashes. Although DRESS syndrome usually manifests with skin lesions, the appearance of myocarditis and pericarditis occasionally occurs in these patients [51]. Data from the literature suggest that the involvement of the myocardium ranges from 13% to 20% [58,59]. In the case of involvement of the heart muscle, symptoms of heart failure most often occur, and, echocardiographically, as many as 86% of patients develop left ventricular dysfunction [60]. Sometimes this type of myocarditis has a self-limiting character, so the disease goes into remission after stopping the drug. However, in a large number of patients, the application of immunosuppressive therapy is necessary. In the case of acute necrotizing eosinophilic myocarditis, the clinical picture rapidly progresses to heart failure, and the mortality in this case reaches up to 55% [60].

### 3.3. Giant Cell Myocarditis

Giant cell myocarditis is a rare type of rapidly progressive myocarditis. The incidence of giant cell myocarditis (GCM) ranges from 0.007% to 0.051% [61]. A large post-mortem study reported that GCM is responsible for approximately 10% of fulminant forms of myocarditis (FM) [62]. Pathohistologically, significant destruction of the myocardium mediated by a large number of cytotoxic T cells, macrophages, giant cells, and eosinophilis was observed within this myocarditis. It is believed that there is an association of this type of myocarditis with immune/allergic processes [63]. It is characterized by sudden onset of HF and refractory malignant ventricular forms of arrhythmia (about 55%) [64]. In the differential diagnosis, it is necessary to distinguish it from myocarditis in sarcoidosis.

### 3.4. Myocarditis Associated with Immune Checkpoint Inhibitors

The use of immune checkpoint inhibitors (ICIs) as anticancer drugs has increased significantly in recent years. As part of their application, an increased incidence of myocarditis was observed, up to 1.9% [36,65]. Acute myocarditis most often occurs in the first month after the start of drug administration [66]. Patients with AM after administration of ICI have a high mortality rate [65,67].

Chimeric antigen receptor T cell (CAR-T) therapy is a new therapeutic approach that modifies T cells to attack cancer cells [68]. This therapy represents the treatment of various hematological malignancies. Cases of myocarditis after the application of this therapy are described in the literature [32,68,69]. When using this drug, as many as 10–15% of patients develop cardiotoxicity, which is manifested by heart failure, arrhythmias, acute coronary syndrome, or cardiovascular death [69].

### 3.5. Myocarditis Associated with Systemic Diseases

The pathogenesis of myocarditis with autoimmune systemic diseases is based on the deposition of immune complexes, the activation of complements, etc. Systemic diseases most often cause the appearance of myocarditis in patients between 25 and 60 years of age. Cardiac lesions are found in 40–60% of autopsy patients who suffered from systemic lupus erythematosus, 50–80% of patients suffering from scleroderma, and in 38% of patients previously suffering from dermatomyositis [46,70].

## 4. Division of Myocarditis

Myocarditis can be classified according to the etiological factor that causes it, the severity of the disease, the dominant symptoms, and the pathohistological findings [1,45]. Sometimes, a combination of the aforementioned criteria classifies the patient in a certain phenotype.

According to the traditional pathohistological Dallas criteria, examination of the biopsy obtained by endomyocardial biopsy (EMB) with a light microscope can initially indicate whether it is **active myocarditis** or **borderline myocarditis**. Active myocarditis is presented by the presence of an inflammatory infiltrate in the myocardium with necrosis and/or degeneration of adjacent myocytes, unlike borderline myocarditis where there is an inflammatory cell infiltrate in the myocardium but without cell necrosis [71].

Based on the histopathological diagnosis of the myocardium and according to the type of cellular infiltrate, we distinguish between lymphocytic, eosinophilic, and polymorphic myocarditis, giant cell myocarditis, and granuloma [45].

According to the etiological factor, myocarditis cases can be divided into those caused by infectious and non-infectious agents. **Infectious myocarditis** is caused by viruses, protozoa, bacteria, fungi, and pathogens that cause direct injury to the myocardium [72]. When myocarditis is caused by a viral infection, the result of which is a direct cytotoxic effect on the myocardium, then it is **virus-mediated myocarditis**. It occurs under the influence of myocardiotropic viruses such as Coxackie virus. **Non-infectious myocarditis** occurs as a result of damage to cardiomyocytes through the direct effect of toxic noxa (drugs, chemicals, vaccines) as a result of hypersensitivity reactions, immune processes, the influence of radiation, or as a part of systemic disease [73,74]. In the case of immune-mediated lymphocytic myocarditis (**virus-triggered myocarditis**), we do not have the presence of a viral genome in the myocardium, but the connection between viral infection and the appearance of AM is clearly correlated (e.g., in the case of influenza) [29]. **Immune-mediated myocarditis** is caused by damage to cardiomyocytes in autoimmune processes (seen in heart transplantation—in cases with alloantigenes), or in systemic diseases (systemic lupus erythematosus, rheumatoid arthritis, Churg–Strauss syndrome, thyrotoxicosis, sarcoidosis, and granulomatosis with polyangiitis—appearing with autoantigens) [45,75,76].

Myocarditis caused by hypersensitivity reactions primarily due to the use of drugs is classified as **hypersensitivity myocarditis (allergic myocarditis)** [75,77]. Myocarditis caused by the direct cytotoxic effect of a drug is called **drug-induced myocarditis [1]**.

Based on clinical features, myocarditis can be divided into AM, chronic myocarditis (CM), and chronic inflammatory cardiomyopathy (CIC) [1]. According to the consensus of experts of the American HF Association, subacute myocarditis (SM) is also included in the clinical division, while in the Japanese Association of Cardiology guidelines, chronic active myocarditis (CAM) and postmyocarditis cardiomyopathy (PMC) are included in the clinical division [1,46]. In both of these documents, inflammatory dilated cardiomyopathy (IDC) is conceptually included in the frame work of HIC. Recent views and consensuses of experts have shown a tendency to classify all myocarditis clinically in one of two forms—AM and CIC [1,46].

### 4.1. Acute Myocarditis

AM is predominantly a clinical diagnosis of myocarditis, which includes a time frame until the diagnosis is made and, if EMB was performed, the presence of findings that are characteristic of active myocarditis. According to the more recent criteria of the American Heart Association (AHA) and the Japanese Association of Cardiology (JAC) AM is a myocarditis in which symptoms began within one month of diagnosis (according to the earlier guidelines of the European Society of Cardiology (ESC), AHA, and JAC, up to three months) [1,45,46,78]. AM is accompanied by an increase in the level of highly sensitive troponin, the presence of myocardial edema diagnosed in CMR, and the infiltration of mononuclear cells with fusion or necrosis of cardiomyocytes proven by the presence of EMB [79,80].

### 4.2. Subacute Myocarditis

SM is a myocarditis variety in which symptoms are present or have worsened since more than three months before the diagnosis (according to the ESC consensus). According to the AHA expert consensus, SM is considered myocarditis when the diagnosis is made between one and three months after the onset of symptoms. According to clinical and histopathological characteristics, it can also be defined as myocarditis in healing, with previously clear evidence of AM [81].

### 4.3. Chronic Myocarditis

CM may represent an intermediate stage between AM and CIC. It is defined as an ongoing inflammatory process with fibrosis, but also without necrosis or abnormality in cardiomyocytes [1,46]. The diagnosis is established if the symptomatology lasts longer than three months before the diagnosis according to the ESC guidelines or longer than one month according to the JAC guidelines [45,46]. The AHA consensus does not clearly define the time frame for CM [1]. The term **chronic active myocarditis** is only described within JAC recommendations and is defined as myocarditis after 30 days from the onset of symptoms with a histopathological picture of active myocarditis [46].

### 4.4. Chronic Inflammatory Cardiomyopathy

CIC indicates a chronic inflammatory condition in which there is a constant infiltration of the myocardium by inflammatory cells, without clear signs of myocardial necrosis [46]. Cardiomyocyte abnormality with local or diffuse fibrosis is expected. This finding is present more than 30 days after the onset of symptoms. Ventricular remodeling accompanied by cardiac failure is present. It can present with dilated cardiomyopathy (inflammatory dilated cardiomyopathy) or without dilated cardiomyopathy (hypokinetic phenotype) [1].

## 5. Clinical Picture

Myocarditis is a disease characterized by polymorphic signs and symptoms. The reason for this lies both in the different etiological factors that lead to myocarditis and in its symptomatology, which is different depending on the stage of the disease in which the patient was first examined. The clinical picture can vary from asymptomatic cases to sudden cardiac death due to cardiogenic shock and malignant rhythm disorders [82]. The disease can occur in patients of all age groups, although it is most common in young people [83].

None of the symptoms in patients with myocarditis are specific to this disease. Apart from the symptoms related to the damage and inflammation of the myocardium itself, the symptoms of other organ systems that can be affected by the infectious agent are also important.

1. **Acute (non-fulminant) myocarditis**—about 65% of patients have this form of myocarditis [84]. This phenotype includes asymptomatic cases and cases in which some degree of cardiac damage may occur with partial or complete regression [36]. These patients have a clinical, electrocardiographic picture and biohumoral syndrome similar to acute myocardial infarction (AMI). In rare cases, death occurs [85].
2. **Fulminant myocarditis (FM)**—accounts for 8.6% of all patients with myocarditis [86]. It is characterized by an acute onset of the disease, which is characterized by rapid deterioration and usually death [36]. Usually, patients present with symptoms and signs of HF (up to pulmonary edema) and not infrequently up to cardiogenic shock, and the clinical course is accompanied by malignant arrhythmias. In these patients, inotropic stimulation or mechanical circulatory support is usually required [46,87]. Patients with FM compared to patients with a non-fulminant form of this disease have a higher early mortality ((28.0% vs. 1.8%, *p* = 0.0001) and late mortality during seven years of follow-up (47.7% vs. 10.4%, *p* < 0.0001) [85]. Earlier studies that monitored the prognosis of patients with FM showed conflicting results regarding the long-term prognosis [88,89,90]. Ammirati E. et al. also showed a correlation between the histological subtype of FM and patient prognosis. Giant cell FM is associated with a significantly worse patient prognosis compared to eosinophilic and lymphocytic subtypes of myocarditis [85].
3. **Chronic active myocarditis** (11% of cases)—its course is similar to AM, with a slower progression of the disease and usually a mild to moderate clinical picture [36]. It is characterized by cardiac dysfunction, occasionally of the restrictive type [36,46].
4. **Chronic persistent myocarditis** (7% of cases)—it is characterized by a mild onset of the disease, usually without cardiac decompensation [36].

### 5.1. Symptoms Related to the Previous Infectious Agent

In the case of viral infectious etiological factors, patients usually have symptoms related to the respiratory and gastrointestinal systems or symptoms similar to a common cold two weeks before the appearance of specific symptoms [91,92]. General symptoms are present in about 80% of patients, and the most common are: chills, fever, elevated body temperature, headache, myalgias, atralgias, fatigue, and sweating. Signs of upper respiratory tract infection (sore throat, cough) and gastrointestinal symptoms (loss of appetite, nausea/vomiting, diarrhea, nonspecific abdominal pain) are not uncommon [86,93,94,95]. According to the HERMES-HF register, 60% of patients with AM have symptoms related to infections of the respiratory system (90%—elevated body temperature, 50%—sore throat, 44.3%—cough) while 15% of patients have gastrointestinal symptoms (100%—diarrheal syndrome, 78%—fever, 56%—abdominal pain, 30%—vomiting) [96].

### 5.2. Chest Pain

In patients who report chest pain at initial presentation, the suspicion of myocarditis can be aroused by the anamnestic data of a respiratory or gastrointestinal infection present 1–4 weeks before the onset of symptoms. Chest pain in the case of myocarditis or myopericarditis occur in 75–95% of patients [95,97,98,99]. The pain may resemble typical angina when it is very important to make a differential diagnosis in relation to AMI [100]. When the pericardium is affected by inflammation, the pain can have different characteristics. The pain is then usually sharp, intensifies during inspiration and forced coughing, and decreases when sitting [101]. In patients with myocarditis, chest pain can also occur as a result of microvascular dysfunction [102]. Spasms of coronary blood vessels caused by inflammation can also be the cause of pain [103]. One of the first descriptions of transient spasms of coronary blood vessels in patients with proven lymphocytic myocarditis was given by McCulli et al. [104].

### 5.3. Symptoms and Signs of Heart Failure

Patients with HF may present first with fatigue, both during normal physical exertion until fatigue and at the slightest physical exertion. In AM, patients have dyspnea and orthopnea in 19–49% of cases [96,105]. Studies have shown that in patients with reduced left ventricular ejection fractions (EF LK < 45%), dyspnea is present in as many as 98.1% of patients, while patients with preserved EF have less dyspnea and the dominant symptom is chest pain [106]. Late inhaling crackles over lung fields are registered during physical examination of the patient. Auscultation of the heart registers the presence of the third heart sound—S3, tachycardia, systolic murmur of mitral and tricuspid regurgitation. In some patients, we have signs (swollen neck veins, pretibial edema) and symptoms of right heart failure. In the most severe cases, the patient will present with a picture of cardiogenic shock, the need for inotropic stimulation, or mechanical support [107,108].

According to data from the multicenter Lombardy registry, about 26% of patients with AM are complicated by the appearance of HF with reduced EF [86]. The results of this registry suggest that patients with AM can be effectively stratified based on their initial clinical presentation. For patients who have HF with EF < 50% (assessed by initial echocardiographic examination), the occurrence of self-sustaining ventricular tachycardia, or the need for inotropes or mechanical circulatory support, it can be said that they have complicated AM with high short-term and long-term mortality (11.9% vs. 18%) [86]. Studies also showed a negative predictive value of the occurrence of HF and the presence of malignant ventricular arrhythmias on the outcome of patients with AM [107,108].

### 5.4. Arrhythmias

The development of arrhythmias in myocarditis is most often a consequence of the development of foci in the sensitive and damaged part of the myocardium [109]. Dysfunction of ion channels, as well as myocardial ischemia, potentiates the electrical instability of cardiomyocytes [110]. In the chronic phase of myocarditis, life-threatening arrhythmias occur due to the development of recurrent myocarditis, residual dysfunction of the left ventricle, and the formation of a post-inflammatory scar of the myocardium. Myocarditis can be complicated by both atrial and ventricular arrhythmias. Different types of potentially life-threatening bradyarrhythmias and tachyarrhythmias can be registered at any stage of the disease as an expression of electrical instability of the myocardium [109,111,112].

Sinus tachycardia is registered in about 26.7–57% of patients [96,113]. Atrial fibrillation is not rare and is registered with a frequency of 3% to 14% [46,114]. It is more often registered in patients with more severe clinical pictures and previous heart diseases [114].

Patients with ventricular rhythm disturbances can have a wide range of symptoms from palpitations (14.4%) and syncope to sudden cardiac death [111,115]. Ventricular arrhythmias are particularly common in patients with FM, especially in patients with GCM (29%) and sarcoidosis (55%) [109].

In the event of conduction disturbances, patients may complain of unsteadiness while walking, dizziness, lightheadedness, or loss of consciousness. Atrioventricular blocks are most often registered in patients with GCM, sarcoidosis, or Lyme disease [116,117,118].

### 5.5. Syncope

Syncope is reported in 6% of patients with myocarditis during the initial presentations [86]. Syncope occurs more often in patients with the fulminant form of the disease [85]. It can be a consequence of the development of severe HF and consequent cardiogenic shock, but it can also occur as a consequence of ventricular rhythm disorders or conduction disorders.

### 5.6. Myocarditis in Children

In about 83% of children’s cases, the diagnosis of myocarditis is not made at the first visit to the doctor, but usually takes two or more visits to the doctor before the diagnosis of myocarditis is made [119]. As in adults, the symptoms and signs of myocarditis can be non-specific. General signs and symptoms such as fever, chills, increased sweating, and loss of appetite may precede. The clinical picture may include dyspnea, vomiting, and the appearance of diarrhea [120]. The occurrence of dysrhythmias is common in the pediatric population with myocarditis and indicates a worse prognosis of the disease [119]. According to the studies of S. Aliaa et al., the largest number of children (76.5%) presents with symptoms and signs of congestive HF without hemodynamic instability, while 11.8% of patients develop a fulminant form of the disease [119].

**Figure 1:**
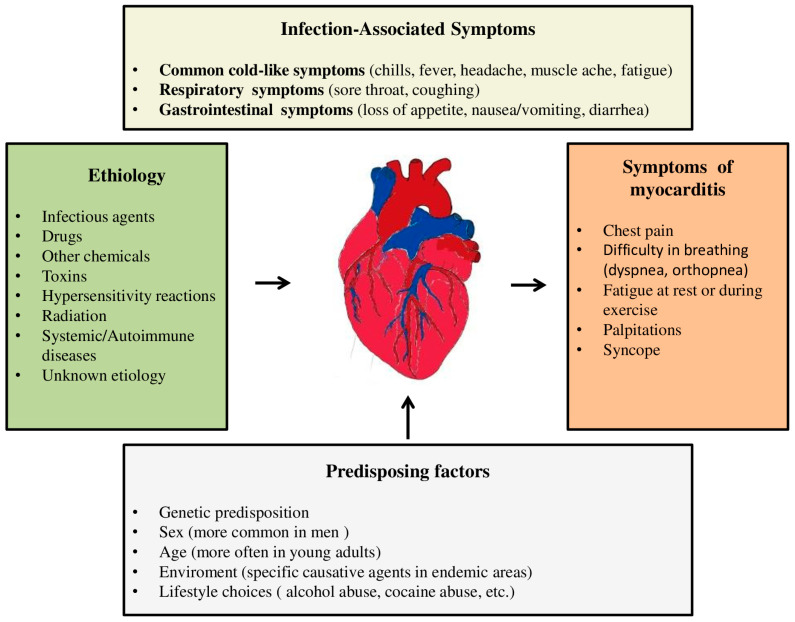
Etiology, predisposing factors, and symptoms of myocarditis.

According to the ESC recommendations, clinically suspected myocarditis requires one or more clinical criteria (acute chest pain or new-onset dyspnea or palpitations/unexplained arrhythmia cardiogenic shock) and ≥1 diagnostic criteria from different categories (ECG features of heart injury, elevated markers of myocardial necrosis, functional/structural abnormalities on echocardiogram/angiogram or CMR) in the absence of an angiographically detectable cause that could explain the existing syndrome [45]. In addition to anamnestic data on previous infection, it is important to pay attention to other potential causes of myocarditis, such as recent exposure to medications, taking drugs, consuming raw meat, or travelling in areas where there are specific viral or bacterial pathogens. Additionally, data on previous diseases and conditions that could be associated with the occurrence of myocarditis are of a great importance (Figure 1).

## 6. Diagnosis of Myocarditis

As myocarditis is a disease with a wide clinical spectrum, from asymptomatic cases to sudden cardiac death, a large number of patients remain undiagnosed. The lack of non-invasive tests with high specificity and sensitivity is another reason why the diagnosis of myocarditis is often missed. According to all guidelines, the most important first point in the diagnostic algorithm is to suspect that the patient has myocarditis, given the polymorphism and non-specificity of the symptoms. Diagnostic includes physical examinations, teleradiography of the heart and lungs, echocardigraphic examination, 24-h ECG Holter monitoring, CMR heart examination, cardiac catheterization, and EMB. Non-invasive diagnostic methods such as CMR can be useful in diagnosing myocarditis and monitoring the progression of the disease. EMB is the gold standard in the final diagnosis of myocarditis; however, not all patients with suspected myocarditis should undergo EMB, but only patients with an unconfirmed diagnosis, especially with a pseudoinfraction image [83,121].

### 6.1. ECG in Myocarditis

Changes in the electrocardiogram can be seen in approximately 90% of patients with AM [95,122]. A 12-lead ECG should be performed in all patients with clinically suspected myocarditis despite its low sensitivity (47%) for myocarditis [36,123]. Both supraventricular arrhythmias (sinus tachycardia and atrial fibrillation/flutter) and ventricular arrhythmias (ventricular extra systoles, ventricular tachycardia, and ventricular fibrillation) are common. Conduction disorders (atrioventricular blocks, bundle branch blocks, and defects in interventricular conduction) are also not rare [124,125,126].

Of all the arrhythmias, sinus tachycardia occurs most often, with variable frequency [127]. Studies suggest that the occurrence of sinus tachycardia is often associated with the development of HF, especially in FM [128]. Repolarization changes at the level of the ST segment and T waves, changes in the height of the R teeth, and the appearance of pathological Q teeth are also often registered in these patients. ST segment elevation is registered more often (prevalence between 24–75%) than ST depression [129]. ST elevation in myocarditis most often occurs when the pericardium is also affected. ST elevation is present in almost all leads in the ECG and is not accompanied by reciprocal ST depression in the contralateral leads (except in aVR and V1) [130]. ST elevation in myopericarditis is concave while in AMI it is convex upwards [131]. Patients with initial ST elevation in the electrocardiogram may develop further evolutionary changes in the form of negative T waves (81.6% of patients), most often on the fourth day after the initial ST segment elevation [128]. However, the evolutionary appearance of negative T waves in the electrocardiogram is not associated with a worse clinical outcome. One condition, which can also be confused with acute perimyocarditis, is benign diffuse ST elevation, called “early repolarization with ST elevation” (ERSTE) [132]. ERSTE is accompanied by diffuse ST segment elevation in the inferior and anterolateral leads. ST depression is present in about 9–18% of patients with acute myocarditis [133,134].

Isolated T wave changes are seen in 9–48% of patients with AM [128,134,135]. Most often, it is an inversion of the T wave. The importance of this change is also reflected in the differential diagnosis, because patients with AMI can also have symmetrical, deep, negative T waves. In a study by De Lazzari et al., it was determined that the presence of negative T waves in the electrocardiogram correlates with the extent of myocardial edema assessed by T2-weighed CMR sequences [136].

Depression of the PR segment can occur in about 2% of cases of myocarditis [4]. It occurs quite often in myopericarditis [137].

Reduction in the amplitude of the QRS complex is seen in about 10% of patients with acute myocarditis [46]. The most common reason for low QRS voltage is the occurrence of pericardial effusion. Nakashima H. et al. showed in their study that 18% of patients with AM have a significant reduction in QRS amplitude during the acute phase of the disease regardless of the presence of pericardia effusion [124]. From a pathophysiological point of view, myocardial edema can be one of the causes of low QRS complex voltage [129,138]. Additionally, a study by Chen J. et al., showed that low QRS complex voltage was present in 44 out of 274 patients and this sign was associated with the occurrence of FM [128]. Low voltage of the QRS complex in the ECG was an independent predictive factor for the occurrence of FM.

The prevalence of a wide QRS complex in AM is variable and depends on the severity of the clinical picture (12–25%) [46,139]. According to some studies, that percentage goes up to 70% in FM [134,140]. A QRS complex of ≥120 msec duration (including the block of the left and right branches of the bundle of His) is an independent predictive marker for FM [85,128]. Ukene et al. pointed out that prolonged duration of the QRS complex is a significant independent predictor of cardiac death or the need for a heart transplant [139]. In a study by Nakashima H. et al., bundle branch blocks of His occurred in 55% of patients with equal prevalence of right and left bundle branch blocks [124,129].

Ventricular tachycardia (VT) is registered in 6.2% of patients with AM, and the results of the study showed that its occurrence is an independent predictive factor of FM and a worse outcome [128]. VT occurs with a much higher frequency in GCM (up to 55%), in EM (11%), and cardiac sarcoidosis (29%) [64,141,142].

The prevalence of first-degree AV block I occurs with a frequency of 4–11% [46,143]. A study by Morger T. et al. showed a prevalence of advanced or complete AV blocks of 15.5% [143]. Ogunbayo G.O. et al. showed that the incidences of cardiogenic shock, respiratory failure, and renal failure were higher in patients with high degree AV blocks compared to patients without conduction disorders (26.2% vs. 5.0%, 33.9% vs. 5.9%, and 29.2% vs. 5.5%, *p* < 0.001, respectively) [125]. A high frequency of AV blocks was seen in Lyme carditis (40%), cardiac sarcoidosis (30%), and GCM (31%) [144,145,146,147]. In their study, Chen J. et al. showed by multivariate logistic regression analysis that independent predictive factors associated with FM were the occurrence of ventricular tachycardia, high degree AV blocks, low QRS complex amplitudes, and QRS complex durations of ≥120 ms. The appearance of pathological Q waves and prolonged QT intervals > 440 ms were also identified as predictors of poor outcome [128].

### 6.2. Biomarkers

Today, a large number of laboratory parameters are used for both diagnostic and prognostic purposes in myocarditis.

**a.**  **Markers of inflammation:** high-sensitivity C-reactive protein (hs-CRP), elevated leukocyte count, and accelerated sedimentation.

Leukocytosis, elevated CRP values, and accelerated sedimentation are always found in patients with myocarditis, but their diagnostic value is low due to their presence in many other diseases [148]. Elevated CRP values and accelerated sedimentation are present in as many as 80–99% of patients [46]. The presence of eosinophilia can be indicated in EM (it occurs in 75.9% of patients) [141]. New inflammatory biomarkers under investigation include the necrosis factor of tumor alpha, interleukin 10, interleukin 6, interferon-g, serum soluble Fas, and soluble Fas ligand levels. Elevation of these markers indicates a worse prognosis, although they are not taken as part of routine laboratory analyses [149,150]. More recent studies have shown elevated levels of heparin-binding protein (HBP) in patients with myocarditis [151]. Additionally, proinflammatory molecules released from monocytes and neutrophils (alarmin S100A8 and S100A9) proved to be significant predictors of myocardial damage, and the highest values in myocarditis were observed in the acute phase of the disease [152]. Serum S100A8/A9 levels in patients with recent-onset myocarditis have been shown to reflect inflammatory disease activity in cardiac tissue independent of viral persistence, age, or sex [152]. Sera soluble ST2 affects the reduction in the proinflammatory activity of IL 33. In a study conducted on 330 male patients younger than 50 years of age with myocarditis, significantly elevated values of sST2 were registered. Elevated values of sST2 correlated with the severity of HF (the same was not proven for patients older than 50 years) [153]. In their studies, Mirna M et al. examined the effect of the ratio of neutrophils and lymphocytes as well as the ratio of neutrophils and monocytes on the severity of myocarditis. Both ratios have been shown to correlate with disease severity followed by longer hospital stay [154].

**b.**  **Markers of myocardial damage**

None of the markers of myocardial damage available so far are specific enough to demonstrate myocardial inflammatory processes. The diagnostic value of these markers varies depending on the time they are taken relative to the onset of the disease. Within AM, the levels of aspartate aminotransferase (AST), lactate dehydrogenase (LDH), creatine kinase (CK-MB), highly sensitive troponins (troponin T and troponin I), and myoglobin are elevated [155,156]. Troponins can be used to demonstrate cardiomyocyte degradation due to myocardial infarction, myocarditis, cardiac arrhythmias, etc. However, on the basis of troponin values, a distinction cannot be made between ischemic and inflammatory cardiomyocyte injury. Elevated troponin values can also be found in other diseases such as aortic dissection, pulmonary thromboembolism, septic injuries, etc. [157]. When it comes to myocarditis, troponin is elevated in at least 50% of patients with proven EMB myocarditis. Most often, troponin values are negative due to the delay in taking a blood sample compared to the onset of the disease. Liu C. et al. examined the absolute and relative changes in hs-cTnI within 24 h and 48 h after admission to the hospital in patients with AM. They showed that absolute changes and relative changes in hs-cTnI within 24 h and 48 h were strong predictors of in-hospital mortality by Cox regression analysis after adjustment for sex, time from onset to admission, and occurrence of VT or VF [158]. Most FM patients who survived experienced a decline in hs-cTnI within 24 h [158]. Today, in the era of ICI use and associated myocarditis, Tn values have been shown to be indispensable for monitoring these patients. High-sensitivity TnI levels are significantly elevated in patients with ICI-induced myocarditis [159]. Early detection of this myocarditis is essential because timely treatment greatly improves the outcome.

**c.**  **Dysfunction markers**

B-type natriuretic peptide (BNP) and N-terminal pro BNP (NT-proBNP) are widely used as diagnostic biomarkers for HF and cardiac dysfunction in clinical medicine. They are released from cardiomyocytes when there are elevated values of ventricular filling pressure. Their measurement in patients with myocarditis and suspected HF is recommended [1]. A more recent study by Sara B. et al. showed that NT-proBNP is significantly correlated with markers of inflammation (leukocyte count and CRP value) in patients with AM [160]. A study by Uken C. and colleagues showed that a high value of NT-pro BNP in patients with myocarditis was predictive for cardiac death in heart transplantations (hazard ratio 9.2; 95% confidence interval 1.7–50; *p* = 0.011) [161].

**d.**  **Anti-cardiac antibodies**

Heart-specific autoantibodies (anti-cardiac autoantibodies) are found in the peripheral blood of patients with myocarditis. The expression of autoantibodies against the heart is the result of the induction of autoimmunity during the process of the immune reaction in order to eliminate the causative agent. In fact, in cases of myocarditis and DCM, there may be anti-cardiac autoantibodies against various tissues, including the contractile structure of the heart (myosin) and the extracellular matrix (laminin) [162]. Autoantibodies against the heart are detected in about 60% of all patients with myocarditis in the chronic phase [18]. In patients who do not have myocarditis, they are detected in 1–3% of cases [163]. Therefore, their use in screening for myocarditis is expected. The presence of anti-cardiac antibodies strongly correlates with the prognosis of patients with myocarditis [164]. In AM, their presence may indicate the risk of cardiac death or the need for a heart transplant [165]. The presence of anti-cardiac antibodies in patients with chronic myocarditis is associated with possible deterioration of cardiac function in the future and transition to DCM.

**e.**  **Micro RNA**

MicroRNAs (miRNAs) are single-stranded, non-coding RNAs that are not translated into protein, act on protein-coding mRNA, and regulate gene expression. An increase in blood mRNAs was observed in patients with AM [166]. Various clinical studies have shown that the levels of multiple miRNAs can be increased or decreased in serum and tissue obtained from patients suffering from myocarditis [167,168,169]. It has been demonstrated that mRNA expression is higher in AM patients than in healthy individuals or in AMI patients. The expression of mRNA allows the differentiation of AM from AMI with an accuracy of ≈93% [46]. miRNA levels are often different in patients suffering from myocarditis compared to patients with other heart diseases, making them promising diagnostic biomarkers [170]. Determination of micro RNA is particularly important in order to obtain gene therapy in the treatment of myocarditis.

**f.**  **Viral antibodies**

In the acute and convalescent phase of the disease, virus titers are positive in less than 40% of cases. The diagnosis of myocarditis cannot be made by determining only the titer of antibodies to cardiotropic viruses in the serum. The diagnosis of possible viral myocarditis is established by the presence of a fourfold increase in the titer of virus antibodies in two samples 3–4 weeks apart with the corresponding clinical picture, echocardiographic findings, and CMR findings. Routine viral serology testing is not recommended.

Today, new biomarkers such as pentraxin 3, galectin 3, and growth differentiation factor are also in use. Total and microribonucleic acid transcriptomic biomarkers promise to improve the diagnostic and prognostic assessment of myocarditis in the future.

### 6.3. Echocardiography

Echocardiography is a standard diagnostic method that should be performed in all patients with suspected myocarditis in order to exclude other causes of HF and reveal the presence of intracardiac thrombi and associated valvular disease. Echocardiography quantifies the degree of systolic and diastolic dysfunction of the left ventricle. It is used to visualize the thickness of the left ventricular wall and to measure the endocavitary dimensions of the chambers. An echocardiographic examination in patients with myocarditis can reveal thicker walls of the left ventricle with abnormal echogenicity of the myocardium due to interstitial myocardial edema, global ventricular dysfunction, segmental outbursts in the kinetics of the left ventricle (especially hypokinesia of the inferior or inferolateral wall of the left ventricle), the presence of diastolic dysfunction of the right ventricle, and the presence of pericardial effusion [171,172]. Thickening of the walls of the ventricle and segmental outbursts in the kinetics are usually transient and last through the acute phase of the disease [46]. Particular attention should be paid to whether segmental breaks in kinetics correspond to the revascularization area of one coronary artery, which is more common in ischemic heart disease and not in myocarditis.

In addition to assessing the function of the left ventricle, it is also important to assess the right ventricle (estimation of its size, systolic excursion of the tricuspid annulus—TAPSE, etc.) [173]. A significant reduction in right ventricular function is a powerful predictor of death and the need for heart transplantation in patients with proven myocarditis [46]. In patients with FM, the dimensions of the heart cavities are normal and the walls are edematous, while in patients with AM, there is pronounced dilatation of the left ventricle with normal thickness of the heart walls. The importance of the finding of reduced EF during the initial examination in patients with myocarditis is reflected in its prognostic significance, which suggests worse patient outcomes [89,174]. The results of a study by Meindl C. et al. showed that the presence of myocarditis in both the acute and subacute phases can be most reliably proven by longitudinal left ventricular strain (LV-GLS), rather than by determining the EF or diastolic volume of the LK by transthoracic echocardiography (*p* < 0.05) [175]. As the differential diagnosis of AM and AMI still represents a great challenge, the application of newer echocardiographic techniques, especially **speckle tracking echocardiography (STE) technology**, has increased accuracy not only in the diagnosis of systolic and diastolic dysfunction of the left ventricle, but also in distinguishing these two diseases [176]. The results of various studies have shown that STE measurements are more sensitive than two-dimensional transthoracic echocardiography in the identification of minor regional disturbances in the kinetics of the left ventricular walls and in the diagnosis of acute viral myocarditis [177,178]. In children and adolescents with FM and normal EF, subclinical abnormality in LK systolic function was proven by 2DE STE examination to correlate with CMR findings of epicardial edema [179]. Additionally, STE can provide significant information about the lack of regional recovery of left ventricular systolic function during follow-up time.

### 6.4. Cardiac Magnetic Resonance (CMR)

Today, CMR is considered the non-invasive gold standard for the diagnosis of myocarditis [180]. CMR is recommended in patients with clinically suspected AM to confirm the diagnosis or in patients with chest pain, normal coronary angiogram, and elevated troponin to resolve the differential diagnosis. It is recommended that CMR be performed in all clinically stable patients [1]. In 2018, the earlier Lake Louise criteria for diagnosing myocarditis were updated [181]. According to these criteria, in patients with high pretest probability, myocardial inflammation is suspected when at least one of the criteria is met: 1. There are positive findings on T2-weighted images or T2 mapping as markers of myocardial edema. 2. There is at least one positive finding among late gadolinium enhancement (LGE), T1 mapping, and ECV as markers of myocardial injury. With the introduction of mentioned novelties, the sensitivity and specificity of CMR for the diagnosis of myocarditis are 87.5% and 96.2%, respectively [182]. However, the diagnostic accuracy may vary depending on the clinical picture, the time of the patient’s examination, and the extent of the necrosis of cardiomyocytes. The study by Francona M. et al. showed that the diagnostic sensitivity of CMR of the heart is high for a clinical picture, similar to myocardial infarction (80%), low for a clinical picture similar to cardiomyopathy (57%), and very low for patients presenting with arrhythmias (40%) [183]. Studies have shown that CMR tissue characterization plays major role in risk stratification in patients with suspected myocarditis [94,184,185]. Progression of LGE and greater extents of focal fibrosis in CMR predict the risk of hospitalization and adverse cardiovascular events in patients with suspected myocarditis [186]. According to the ITAMY study, the anteroseptal accumulation of LGE in the midwall layer of patients with AM is associated with a worse outcome compared to other patterns of presentation [98,187]. Additionally, the study by Gräni C. and associates showed that regarding location and pattern, septal and midwall LGE showed strongest associations with MACE (HR: 2.55; 95% CI: 1.77 to 3.83 and HR: 2.39; 95% CI: 1.54 to 3.69, respectively; both *p* < 0.001) [94].

It is best to use CMR to identify acute, active inflammation of the myocardium, and the highest sensitivity is achieved if the examination is performed within 2–3 weeks of the onset of symptoms [1,188]. Myocardial edema is best seen on T2 imagining. CMR can be repeated during patient follow-up, usually after 6–12 months, in order to identify post-inflammatory scars.

The disadvantages of CMR are that it is difficult to perform in patients who cannot hold their breath for a long time or who are hemodynamically unstable, as well as in patients on mechanical ventilation or other intracorporeal devices. Claustrophobia is also one of the contraindications for the application of this modern diagnostic method. Although the importance of performing CMR in AM has been widely demonstrated, this technology remains underutilized, in part due to the limited availability of CMR in standard clinical practice [189].

### 6.5. FDG–PET Scan

Consumption of 18F-fluorodeoxyglucose (18F-FDG) is a quantitative surrogate parameter of increased glucose metabolism, which is a hallmark of the inflammatory process. The use of PET–CT in various forms of myocarditis caused by sarcoidosis, viruses, GCM, and post-infraction myocarditis has long been known [171,190,191,192]. 18F-FDG PET proved to be important both in establishing a diagnosis and in monitoring the response to the application of therapeutic regimens, especially in patients with cardiac sarcoidosis [193]. A study by Nensa F. and colleagues showed that the use of FDG–PET in the diagnosis of myocarditis has a sensitivity of 74% and a specificity of 97% [194]. Novel PET radiopharmaceuticals with affinities for somatostatin receptors, such as the 68Ga-lebeled peptides DOTATOC, DOTATATE, and DOTANOC, have been shown to have a higher diagnostic accuracy than 18F-FDG for cardiac sarcoidosis [195]. Some studies have shown that the sensitivity and specificity of FDG-PET and CMR in the diagnosis of myocarditis show strong complementarities [196,197]. One of the potential advantages of PET-CT over CMR is that it can quantify the degree of inflammation [171].

### 6.6. Endomyocardial Biopsy (EMB)

Endomyocardial biopsy (EMB) has long been considered the gold standard, but has variable sensitivity and specificity [45]. Although EMB is often indicated for the diagnosis of myocarditis, the sensitivity of this method is debatable given the limited possibility of myocardial sampling. Currently, the only way to identify and characterize the inflammatory cell infiltrate is to perform EMB. According to the Dallas criteria, acute myocarditis is defined by lymphocytic infiltrates associated with myocyte necrosis [71,198]. Borderline myocarditis is characterized by inflammatory infiltrates without the presence of myocyte necrosis. Histological criteria of myocarditis are present in only 5–30% of patient with clinically suspected myocarditis and up to half of patients with DCM. EMB provides unique useful information related to diagnosis and prognosis, and its results help in the selection of therapeutic modalities. However, the use of EMB has decreased over time, probably due to its invasive nature, so only its rational application gives an optimal result. EMB is of particular importance in patients with an acute clinical pictures and in life-threatening cases requiring rapid diagnosis and adequate treatment [198]. Although EMB in a large number of cases confirms the diagnosis of myocarditis, its results should be interpreted in the context of (1) clinical probability before testing, (2) sample collection time, (3) sample collection quality, (4) sample collection site, (5) historical type of myocarditis, and (6) analytical methods that are applied [1].

Immunostaining is a diagnostic modality that is very important in the diagnosis of myocarditis. This technique uses antibodies to detect and quantify antigen levels in patients. This method can detect changes in the tissue of a myocarditis patient before myocardial infiltration by inflammatory cells or myocytolysis become histologically detectable [199]. Thus, in addition to hematoxylin–eosin staining, immunostaining [CD3, CD 68, major basic protein (MBP), tenascin C (4C8), tenascin C (4F10)] is useful for histological diagnosis [46]. It has been shown that the level of tenascin C expression is correlated with histologically proven inflammatory activity, suggesting that tenascin C may be a useful marker for assessing disease activity in myocarditis [200]. Immunostaining is also an important method for proving myocarditis in patients suffering from COVID-19 infection, in which a significant increase in CD 68 lymphocytes is registered [201].

## 7. Differential Diagnosis

**Figure 2:**
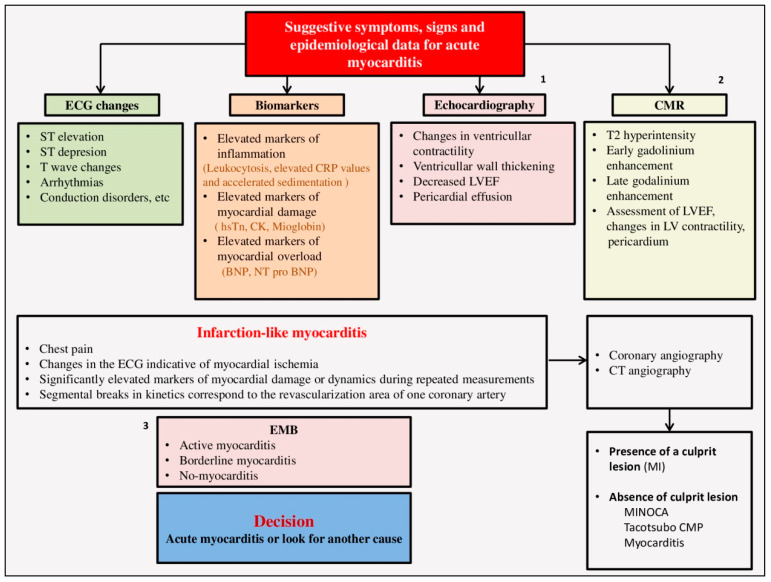
**Diagnostic algorithm for acute myocarditis.** 1. If there are possibilities, speckle tracking echocardiography (STE) technology should be performed; 2. CMR of the heart can only be done in hemodynamically stable patients; 3. EMB is indicated in cases of severe forms of the disease. Abbreviations: ECG, electrocardiogram; CRP, C-reactive protein; hsTn, highly sensitive troponin; CK, creatine kinase; BNP, brain natriuretic peptide; NT pro BNP, N-terminal prohormone of brain natriuretic peptide; LVEF, left ventricular ejection fraction; CMR, cardiac magnetic resonance; LV, left ventricle; CT, computerized tomography; EMB, endomyocardial biopsy; MI, myocardial infarction; MINOCA, myocardial infarction with non-obstructive coronary arteries; CMP, cardiomyopathy.

The most difficult differential diagnosis of AMI occurs when the patient presents with severe chest pain, ECG changes, elevated markers of cardiac necrosis, and heart failure. For patients with myocarditis who have chest pain and changes in the ECG, the term **infarction-like myocarditis** was introduced [1]. According to the registries, the clinical picture occurs in up to 45.8% of patients with myocarditis. According to a prospective observational study by Pasupathy S. et al., it was shown that in as many as 33% of patients who were initially recognized as having a myocardial infarction without coronary vessel obstruction, the underlying cause was myocarditis [202]. In the differential diagnosis, we must take into account that patients with myocarditis are usually younger and that the presence of a history of recent viremia, slow-developing ECG changes involving more than one vascular territory, the lack of reciprocal ST depression, and echocardiographic disturbances in kinetics are global rather than segmental. Additionally, ST elevation in myocarditis is usually transient and can disappear within 24 h (49%) or 48 h (74%) [129]. If there is still a differential diagnostic dilemma between these two diseases, it is necessary to perform cardiac catheterization [45]. Coronary angiography is usually normal in myocarditis, and if there is still a dilemma, it is removed by EMB. Patients with acute myocarditis and acute pericarditis may complain of similar symptoms. It is sometimes difficult to make a differential diagnosis between acute myocarditis and **non-ischemic cardiomyopathy** (Takotcubo cardiomyopathy) or drug-induced cardiomyopathy [203,204] (Figure 2).

## 8. Treatment

Spontaneous recovery of left ventricular function is common in patients with acute myocarditis. It is necessary to treat such patients symptomatically because specific treatment options have not yet been found. Autoimmune myocarditis (large cell myocarditis) is treated with immunosuppressive therapy. A smaller number of patients require mechanical circulatory support or heart transplantation within a year. The prognosis of patients treated for myocarditis is variable, with a five-year survival rate of 60–90% [205].

**(A)**  **Classical treatment**

**Hemodynamically stable patients**

Patients with myocarditis and reduced EFLK are treated according to current guidelines for the treatment of HF [206]. Hemodynamically stable patients with AM and developed HF should be treated with diuretics, ACE inhibitors, or AT receptor blockers and beta adrenergic blockers. Treatment should be continued at least six months in patients with reduced LVEF, even if LVEF improves (>50%), and according to guideline-guided recommendations [45,207]. Nonsteroid anti-inflammatory drugs, especially acetylsalicylic acid, should not be used in myocarditis accompanied by HF because their use is associated with an increase in mortality [45]. They are reserved in the smallest possible dose for patients with perimyocarditis. Recent case-control studies show that their use is not associated with increased mortality in patients with myocarditis [208,209].

**Hemodynamically unstable patients**

Hemodynamically unstable patients with HF should be treated in intensive care units in accordance with existing recommendations for the treatment of HF. In acute/fulminant cases with cardiogenic shock and severe ventricular dysfunction, administration of inotropic and vasopressor drugs is necessary [87,210]. In the event that the intravenous administration of inotropes does not lead to hemodynamic improvement of the patient, and the signs and symptoms of shock are still maintained, the use of mechanical circulatory support is advised for the purpose of bridging until healing or heart transplantation [87]. Heart transplantation should be postponed in the acute phase due to possible cures, but should be considered in hemodynamically unstable patients with GCM.

**Arrhythmias**

There are no special recommendations for the treatment of arrhythmias in myocarditis, so the treatment should be according to current recommendations [211]. Malignant arrhythmias should be treated with antiarrhythmias. Implantation of an implantable cardioverter defibrillator should be delayed in an acute episode of myocarditis and considered usually three to six months after the onset of the acute phase of the disease. Patients with GCM and malignant ventricular arrhythmias who have a life expectancy without heart transplantation of >1 year should receive an ICD [45]. In cases of complete atrioventricular blocks, it may be necessary to install a temporary pacemaker [83]. Atrioventricular blocks are transient, and in many cases resolves within about one week of starting treatment [45,212]. Immunosuppressive therapy with steroids can promote recovery of atrioventricular nodal conduction. In some patients, however, complete atrioventricular blocks may persist even after the acute phase, requiring permanent pacemaker implantation [213]. Resynchronization therapy and the application of mechanical pumps enable longer survival of patients with severe DCM. The indications for implantation of an ICD and cardiac resynchronization defibrillator (CRT-D) should be considered in the chronic phase of the disease to prevent sudden cardiac death, even in recovering patients, if ventricular arrhythmia persists [214]. Primary prevention of sudden cardiac death is based on the SCD–Heft study criteria: chronic heart failure, NYHA > II, and an left ventricular ejection fraction (LVEF) < 35% [215].

**Avoidance of intensive physical activities**

In the acute phase of myocarditis, intense physical activity is prohibited until the disease is completely cured (at least six months) [216]. Athletes should be temporarily excluded from professional sports activity regardless of age, sex, severity of symptoms, or therapeutic regimen.

**(B)**  **Immunomodulatory therapy**

**Antiviral therapy**

Antiviral therapy can theoretically be effective for the treatment of viral myocarditis because viral infection is an integral part of the pathological process. For example, interferon-b may be effective in treating myocarditis caused by enterovirus (Coxsackie virus) or adenovirus infection, and acyclovir may be effective in treating myocarditis caused by human herpes virus infection [45,217,218]. In Murino Coxsacke virus B3-induced myocarditis, interferon beta and alpha therapy protects myocytes from damage and reduces the inflammatory response [219].

**High doses of intravenous immunoglobulins**

Intravenous immunoglobulins are a source of passive immunity and help to clear the virus. Intravenous administration of high doses of immunoglobulin leads to faster recovery of left ventricular function and higher survival during the first year of the disease. Immunoglobulins should be used in patients with DCM whose left ventricular function is progressively deteriorating despite full drag therapy [220,221]. A multicenter study by Kishimoto C. et al. showed that IVIG 1–2 g/kg for two days significantly increased the one-month survival rate and significantly reduced cytokines, including TNF-a and IL-6 [221]. An American observational study showed that high-dose IVIG can significantly increase LVEF in patients with myocarditis with EF LK (LVEF) < 30% [220].

**Immunoabsorption**

The basic principle of immunoabsorption is to remove cardiotoxic autoantibodies from the patient’s plasma by plasmapheresis [222]. The complete amount of IgG antibodies found in the plasma is absorbed during repeated cycles at certain time intervals (daily during the first three days, and then four cycles with an interval of one month). After each session, plasma IgG must be replenished by an injection of 0.5 g/kg polyclonal IgG.

**(C)**  **Immunosuppressive therapy**

Patients with DCM and immunohistologically proven chronic inflammation, but without persistent viral infection, benefit from immunosuppression. A six-month immunosuppressive treatment in these patients can lead to a significant improvement in HF symptoms. Immunosuppressive therapy may be considered when patient has a relapse, recurrent exacerbation, or persistent infiltration of inflammatory cells (e.g., when chronic active myocarditis is suspected) [46]. Patients with EM respond particularly favorably to immunosuppressive therapy, with increased survival rates [223]. Currently, no evidence is available to support the efficiency of immunosuppressive therapy for acute lymphocytic myocarditis. Patients with DCM and persistent infection with cardiotropic viruses should not undergo immunosuppression, as they are candidates for antiviral treatment. So far, treatment with immunosuppressive drugs has shown controversial results. The use of azathiopyrine and prednisone in patients with chronic DCM has been shown to improve left ventricular function, while in GCM combined treatment with immunosuppressants (cyclosporine and corticosteroids with or without azathiopyrine or muronomab-CDs) can extend the average survival time to twelve months compared to untreated patients, whose average survival time is three months [83]. Patients treated with tacrolimus (target serum concentration, 8–12 ng/mL in short-term treatment and 6–8 ng/mL in long-term treatment) have fewer adverse reactions that those treated with the old immunosuppressive regimen [45].

In the case of proven myocarditis associated with DRESS, patients should be treated with rapid administration of high-dose steroids. Unlike GCM, eosinophilic myocarditis responds well to pulse therapy with steroids. Azathioprine can be used as a drug of second choice. There are also described cases of a good response to the application of target therapy in the case of EM. The oral JAK1/JAK3 inhibitor tofacitinib shows strong anti-eosinophilic activity and has been shown to be a good choice in the case of myocarditis associated with DRESS syndrome [224]. Additionally, case reports show that the administration of mepolizumab (anti-IL-5 recombinant humanized monoclonal antibody) can be effective in improving cardiac function in patients with EM. Mepolizumab can also be used as an adjunct to steroid therapy in EM [225]. Tocilizumab is a monoclonal antibody that antagonizes the interleukin-6 (IL-6) receptor and is approved for the treatment of severe forms of COVID-19 infection [226]. The trial showed that a combination of tocilizumab and favipiravir significantly reduces the inflammation caused by the cytokine storm [227]. Additionally, its use in the context of COVID-19 myocarditis was associated with both a shorter length of hospitalization and a lower mortality in these patients [226].

## 9. Prognosis

The mortality rate in patients with myocarditis is still high despite advances in diagnostic and therapeutic modalities (one-year mortality is around 20% and five-year mortality is 50%) [7]. Studies have shown that mortality from acute non-fulminant myocarditis is about 8–12%. Study data from the registry of the Spanish National Health Service show that hospital mortality in patients with AM is 3% (data analyzed from 2003 to 2015) [228]. Predictors of mortality in these patients were shown to be: decreased EFLK, presence of syncope at initial presentation, right ventricular dysfunction, presence of pulmonary hypertension, and prolongation of the QRS complex for more than 120 s. Extremely elevated levels of IL10 as well as soluble Fas have been shown to be predictors of poor outcome in patients with FM. Although up to 80% of patients with AM have normalized left ventricular size and systolic function, the incidence of DCM among patients with histologically positive AM ranges between 14% and 50%. When it comes to children, after acute myocarditis, about 21% of patients develop DCM [229]. Long-term mortality (up to 90 days of follow-up) after myocarditis is about 4.9% [111].

## 10. Conclusions

Myocarditis is still a disease with high mortality and morbidity, especially in the young population. Establishing an accurate and timely diagnosis in myocarditis is still challenging due to the huge number of etiological agents and associated diseases that in their natural course can lead to this disease.

However, by improving diagnostic modalities and searching for increasingly sensitive biomarkers (S100A8/A9, sST2, anti-cardiac antibodies, miRNA, pentraxin 3, galectin 3, and growth differentiation factor), we are getting closer to the possibility of early non-invasive diagnostic and adequate monitoring of these patients. In hemodynamically stable patients, the gold standard in diagnosis of this disease is CMR, which should be used more, i.e., to work on its greater availability in standard clinical practice. EMB is the main invasive diagnostic method, especially in patients with fulminant forms of the disease. Although only this diagnostic method can identify and characterize the infiltrate of inflammatory cells, its use has decreased over time, probably due to its invasive character; consequently, only its rational application provides an optimal result. It is necessary to perform early risk stratification in patients with myocarditis and single out the most vulnerable group of patients in order to prevent possible adverse events and provide an adequate therapeutic regimen. Reduced EF at initial presentation and the occurrence of malignant ventricular arrhythmias with circulatory collapse are significant predictors of poor prognosis in patients with myocarditis.

New therapeutic modalities, the development of immunomodulatory and immunosuppressive therapy, improving the education of doctors in the field of mechanical circulatory support, and timely heart transplantation are just some of the therapeutic aspects that will improve the survival of these patients. Large additional tests are still needed both in the field of diagnostics and in the field of therapy in order to improve treatment, prevent the transition from an acute to a chronic forms of the disease, prevent the recurrence of the disease, and ultimately reduce the mortality of these patients.

## Data Availability

Not applicable.
